# Safety of carotid endarterectomy in the elderly and octogenarian population: a nationwide study including 80,000 patients

**DOI:** 10.1007/s10143-026-04174-4

**Published:** 2026-02-14

**Authors:** Victor Gabriel El-Hajj, Joanna M. Roy, Basel Musmar, Wi Jin Kim, Michael Rizzuto, Nathaniel Ellens, Rabab Alshahrani, Victor E. Staartjes, Adrian Elmi-Terander, Ramachandra P. Tummala, Stavropoula Tjoumakaris, M. Reid Gooch, Robert H. Rosenwasser, Ziad Khabbaz, Pascal Jabbour

**Affiliations:** 1 Department of Neurological Surgery, Thomas Jefferson University Hospital, 901 Walnut street 3rd floor, Philadelphia, PA 19107 USA; 2 Department of Clinical Neuroscience, Karolinska Institutet, Stockholm, Sweden; 3 Trillium Health Partners, 100 Queensway West, Mississauga, ON Canada; 4 Machine Intelligence in Clinical Neuroscience (MICN) Laboratory, Department of Neurosurgery, University Hospital Zurich, Zurich, 8091 Switzerland; 5 Department of Neurosurgery, University of Minnesota Twin Cities, Minneapolis, MN USA; 6 Department of Cardiovascular Medicine, Hotel-Dieu De France, Achrafieh, Beirut, Lebanon

**Keywords:** Carotid stenosis, Carotid endarterectomy, Elderly, Octogenarians, Safety

## Abstract

**Introduction:**

Carotid endarterectomy (CEA) is an established procedure for stroke prevention in patients with carotid artery stenosis. While CEA is considered safe in younger patients, perioperative risks in octogenarians remain debated, with current guidelines classifying the procedure as “high-risk” in this patient population. This study aimed to evaluate short-term outcomes of CEA across age groups and to assess whether comorbidity burden better predicts outcomes than chronological age.

**Methods:**

The ACS-NSQIP database (2013–2020), was used to identify patients eligible for inclusion. The cohort was stratified based on age < 60, 60–80, and > 80 years. Propensity score matching and multivariable logistic regression were used to compare outcomes across age groups and assess predictors of 30-day complications, readmission, reoperation, non-home discharge, and mortality. Interaction analyses were performed to evaluate the combined impact of age, functional status and comorbidity (ASA classification) on outcomes.

**Results:**

Of 82,427 patients, 15,111 (18%) were > 80 years. Octogenarians had significantly higher 30-day complication, readmission, reoperation, non-home discharge, and mortality rates compared with patients aged 60–80 (all *p* < 0.001), even after propensity matching. Logistic regression confirmed increased risk in octogenarians (aOR 1.34, 95% CI 1.27–1.42), but comorbidity burden and functional dependency were stronger predictors; severe comorbidity (ASA 4–5; aOR 2.17, 95% CI 1.91–2.47) and full dependency (aOR 2.61, 95% CI 1.89–3.59). Interaction analysis demonstrated that octogenarians with low comorbidity had risks comparable to younger patients with moderate comorbidity.

**Conclusions:**

CEA is associated with a worse risk profile among octogenarians. Nonetheless, comorbidity burden and functional status are stronger predictors of adverse outcomes, as compared to age alone. CEA can be performed safely in carefully selected octogenarians with low to moderate comorbidity, whereas severe comorbidity or dependency may represent relative contraindications. Surgical candidacy should be guided by physiological reserve and function rather than chronological age alone.

**Supplementary Information:**

The online version contains supplementary material available at 10.1007/s10143-026-04174-4.

## Introduction

As the world’s population continue to age, the number of octogenarians and nonagenarians undergoing surgical interventions is steadily rising [5, 7]. Carotid endarterectomy (CEA) remains a cornerstone in the prevention of ischemic stroke for patients with carotid artery stenosis [3]. 

While CEA is generally considered safe in younger and moderately elderly patients, concerns persist regarding perioperative morbidity and mortality in the very old, with mixed data being previously published [11, 16, 22]. 

For instance, the European Society for Vascular Surgery 2023 guidelines classify patients over 80 as “high-risk” for CEA, indicating that these patients may not gain long-term stroke-prevention benefits from surgery [15]. However, these guidelines are based on data that are nearly two decades old. Oppositely, more recent evidence suggests that CEA in elderly patients is associated with only a modest increase in perioperative risk [11]. These findings are consistent with recent evidence demonstrating marked decreases in adverse outcomes after CEA in contemporary practice [11, 20]. These advances in operative and perioperative care, along with the steadily aging population, have prompted several researchers to reconsider whether CEA should still be considered a high-risk procedure in patients over 80 years of age.

The current state of the litterature with some studies suggesting acceptable perioperative risk in octogenarians, and others indicating elevated rates of complications and death, leave an evidence gap that complicates clinical decision-making. To address this gap, we aimed to compare short-term (30-days) postoperative outcomes after CEA across different age groups (< 60, 60–80, and ≥ 80 years). Furthermore, we sought to explore whether baseline comorbidity burden, as measured by the American Society of Anesthesiologists (ASA) classification, provides a better prediction of adverse outcomes than chronological age alone.

## Methods

### Patients population

ACS-NSQIP is a national US database intended to measure quality of surgical care. Across the country, there are over 600 participating centers enrolled in the database, which provide prospectively collected representative data on surgical procedures and associated outcomes.

All CEA cases (CPT code of 35301*)* entered in the ACS-NSQIP between 2013 and 2020 were eligible for inclusion [18]. This study was compliant with the Strengthening the Reporting of Observational Studies in Epidemiology (STROBE) guidelines. The need for Institutional Review Board approval was waived and there was no need for patient consent as the study used deidentified data from a national registry. The study was performed in accordance with the Declaration of Helsinki and all other relevant ethical guidelines.

## Variables of interest and primary outcomes

All variables in the ACS-NSQIP are defined in an accompanying user guide. Baseline and demographics characteristics including age, sex, BMI, race, ASA classification, functional status, admission origin, and comorbidities were retrieved. Comorbidities in the NSQIP including chronic steroid use, ventilator-dependent; disseminated cancer; history of diabetes, hypertension; congestive heart failure; dyspnea; smoking; COPD; dialysis and acute renal failure, were also recorded. Outcomes were recorded within 30 days after surgery and included: (1) wound complications, encompassing both superficial and deep surgical site infections as well as wound dehiscence; (2) other infections, including pneumonia, urinary tract infection, and sepsis; (3) thromboembolic events, including pulmonary embolism and deep vein thrombosis; (4) unplanned intubation; (5) stroke; (6) myocardial infarction (MI); and (7) bleeding. A composite outcome was defined as the occurrence of any of the aforementioned complications. Additional outcomes included non-home discharge, 30-day readmission, reoperation, and mortality.

### Statistical analysis

Continuous data are presented as means and standard deviations (SD), and categorical data as frequencies (n) and proportions (%). A 1:1 propensity score-based matching protocol using the K-nearest neighbor (KNN) approach with replacement and a caliper of 0.05 was used. Patients were matched based on the year of admission, sex, race, height, weight, BMI, inpatient vs. outpatient treatment, and emergency vs. elective setting, ASA class, functional status, smoking status, dyspnea, diabetes, congestive heart failure, hypertension, chronic obstructive pulmonary disease, renal failure, ascites, chronic steroid use, preoperative wound infection, transfusion status, preoperative sepsis, previous history of cancer. Propensity score matching was evaluated using standardized mean differences and Love plots. A multivariable logistic regression model was created to identify the variables that predict 30-day adverse outcome including complications, readmissions, reoperations, and deaths following CEA. The model was adjusted for all available baseline covariates. To further examine the impact of other variables such as functional status and ASA class on the relation between age and outcomes, an interaction analysis with interaction plots were created. ASA classes were grouped into low (ASA 1–2), intermediate (ASA 3), and high comorbidity burden (ASA 4–5), as previously described [6]. The cohort of patients was initially divided in three age groups 1) < 60, 2) 60–80, and 3) > 80, in accordance with previous literature [1]. In the matched analysis, when specifically studying outcomes among octogenarians (group 3), controls consisted of patients from the closest age group (group 2). Outcomes were compared using Chi-square, Fisher exact, and t-tests as appropriate, with statistical significance evaluated at *p* < 0.05. Statistical analysis was performed using R (version 4.5.1) [4]. 

## Results

### Baseline characteristics

The study cohort included 82,427 patients undergoing CEA (Table 1), with 9,590 (12%) aged < 60 years, 57,726 (70%) aged 60–80 years, and 15,111 (18%) aged > 80 years. Males comprised 61% of the cohort, with a slightly lower proportion in the youngest group (57%) compared to the other age categories (60–62%; *p* < 0.001). Mean BMI differed across groups, being highest in the < 60 group (30.0) and lowest in the > 80 group (27.2; *p* < 0.001).

**Table 1:** Baseline characteristics of the initial cohort

Variable	Overall, *N* = 82,427	< 60, *N* = 9,590	60–80, *N* = 57,726	> 80, *N* = 15,111	*p*-value
Age	70.9 (70.8, 70.9)	54.7 (54.6, 54.8)	70.3 (70.2, 70.3)	83.6 (83.5, 83.6)	**< 0.001**
Male sex	50,036 (61%)	5,484 (57%)	35,506 (62%)	9,046 (60%)	**< 0.001**
BMI	28.8 (28.7, 28.8)	30.0 (29.8, 30.1)	29.1 (29.0, 29.1)	27.2 (27.1, 27.2)	**< 0.001**
Race					**< 0.001**
Asian	1,580 (1.9%)	173 (1.8%)	1,086 (1.9%)	321 (2.1%)	
Black	4,107 (5.0%)	688 (7.2%)	2,859 (5.0%)	560 (3.7%)	
White	76,740 (93%)	8,729 (91%)	53,781 (93%)	14,230 (94%)	
Smoker	23,263 (28%)	5,674 (59%)	16,504 (29%)	1,085 (7.2%)	**< 0.001**
Comorbidity burden					**< 0.001**
Low (ASA 1–2)	3,642 (4.4%)	546 (5.7%)	2,596 (4.5%)	500 (3.3%)	
Intermediate (ASA 3)	60,898 (74%)	7,127 (74%)	42,897 (74%)	10,874 (72%)	
Severe (ASA 4–5)	17,887 (22%)	1,917 (20%)	12,233 (21%)	3,737 (25%)	
Transferred from					**< 0.001**
Home	77,173 (94%)	8,847 (92%)	54,383 (94%)	13,943 (92%)	
Acute care hospital	2,296 (2.8%)	336 (3.5%)	1,460 (2.5%)	500 (3.3%)	
Emergency department	1,887 (2.3%)	317 (3.3%)	1,231 (2.1%)	339 (2.2%)	
Nursing home - Chronic care - Intermediate care	733 (0.9%)	57 (0.6%)	425 (0.7%)	251 (1.7%)	
Other	338 (0.4%)	33 (0.3%)	227 (0.4%)	78 (0.5%)	
Functional status					**< 0.001**
Independent	79,966 (97%)	9,362 (98%)	56,249 (97%)	14,355 (95%)	
Partially dependent	2,283 (2.8%)	209 (2.2%)	1,366 (2.4%)	708 (4.7%)	
Totally dependent	178 (0.2%)	19 (0.2%)	111 (0.2%)	48 (0.3%)	

Racial distribution varied significantly between age groups (*p* < 0.001), with the majority being White (93%), and small proportions of Asian (1.9%), and Black (5.0%) patients. The prevalence of smokers was substantially higher among patients < 60 years (59%) compared with 29% and 7% in the 60–80 and > 80 groups, respectively (*p* < 0.001). Comorbidity burden differed across age groups (*p* < 0.001), with the majority of patients demonstrating an intermediate comorbidity burden (ASA 3; 72–74%) and a higher proportion of severe burden (ASA 4–5) in the oldest group (25%).

Regarding preoperative status, most patients were independent in functional status (95–98%), and the majority were transferred from home (92–94%).

### Rate of 30-day adverse events prior to propensity score matching

**Fig. 1:**
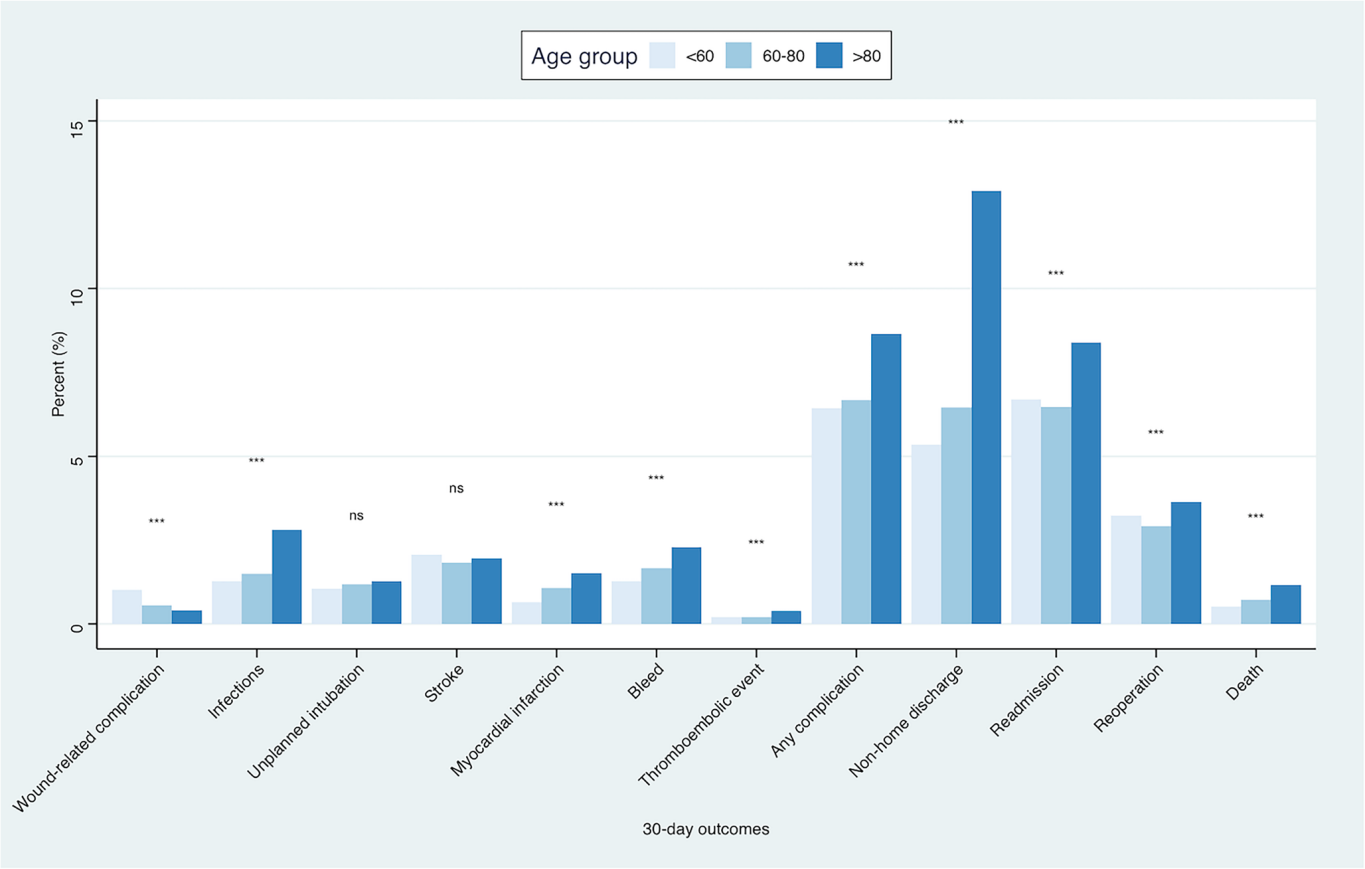
Bar plots showing the rate of different adverse events at 30-days postoperatively among the different age groups

Analysis of 30-day postoperative outcomes revealed that the incidence of most complications increased with age (Fig. 1). Overall, the composite complication outcome combining any complication, including infections, myocardial infarctions, thromboembolic events, and bleedings occurred more frequently in patients > 80 years compared to younger groups (*p* < 0.001). Non-home discharge was also notably higher in the oldest cohort, with more than double the rate observed in patients < 60 years (*p* < 0.001). Similarly, rates of readmission and reoperation were elevated in the > 80 age group (*p* < 0.001). Finally, mortality within 30 days was higher among patients > 80 years compared to the other age groups (*p* < 0.001).

### Rate of 30-day adverse events following propensity score matching

**Fig. 2:**
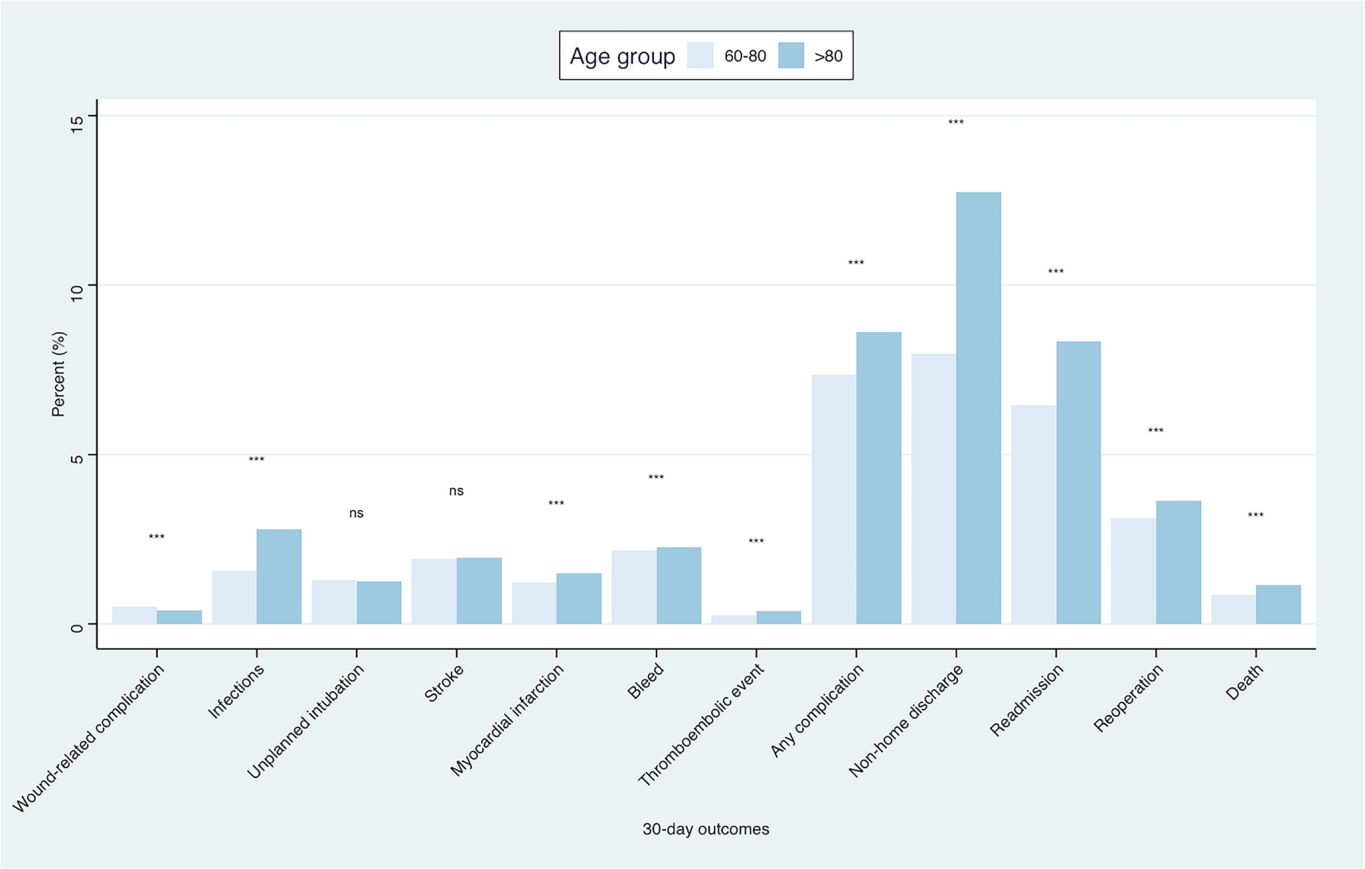
Bar plots showing the rate of different adverse events at 30-days postoperatively among the two age groups, following propensity score matching

Following 1:1 propensity score matching contrasting patients aged 60–80 with those over 80, a total of 15,064 patients were included in each group. Satisfactory matching was achieved as demonstrated by the Love plot (Supplementary Figure A). Similar findings were obtained, with octogenarians (> 80 years) demonstrating significantly higher rates of any complication (*p* < 0.001), as well non-home discharges (*p* < 0.001), readmissions (*p* < 0.001), reoperations (*p* < 0.001), and deaths (*p* < 0.001) during the 30-day postoperative period (Fig. 2).

### Predictors of 30-day adverse events

Multivariable logistic regression was performed to evaluate predictors of any 30-day adverse outcome, including complications, readmissions, reoperations, and death after CEA (Table 2). Compared with patients aged 60–80 years, those over 80 years had a significantly higher risk of adverse outcomes (aOR 1.34, 95% CI 1.27, 1.42, *p* < 0.001), while patients younger than 60 years did not differ significantly from the reference group (OR 0.95, 95% CI 0.88, 1.02, *p* = 0.13).

**Table 2:** Multivariable logistic regression for the prediction of any 30-day adverse outcome including complications, readmissions, reoperations, and deaths following CEA

Variables	aOR*	95% CI	*p*-value
**Age group**			
< 60	0.95	0.88, 1.02	0.13
60–80	Reference	Reference	—
> 80	1.34	1.27, 1.42	**< 0.001**
**ASA class**			
1–2	Reference	Reference	—
3	1.34	1.19, 1.52	**< 0.001**
4–5	2.17	1.91, 2.47	**< 0.001**
**Functional status**			
Independent	Reference	Reference	—
Partially dependent	1.64	1.47, 1.82	< 0.001
Totally dependent	2.61	1.89, 3.59	< 0.001

*Adjusted ORs using all available baseline covariates

Higher ASA classification was strongly associated with adverse outcomes. Patients with ASA class 3 had 34% increased odds compared with those in ASA class 1–2 (aOR 1.34, 95% CI 1.19–1.52, *p* < 0.001), while those with ASA class 4–5 had more than a twofold increase in odds (aOR 2.17, 95% CI 1.91, 2.47, *p* < 0.001).

A decreased functional status was also strongly associated with the occurrence of adverse outcomes. Notably patients who were partially or fully dependent had significantly higher odds as compared to those who were independent (aOR 1.64, 95% CI 1.47, 1.82, *p* < 0.001 and aOR 2.61, 95% CI 1.89, 3.59, *p* < 0.001; respectively).

### Interaction of age and ASA class and impacts on outcomes

The rates of non-home discharge and adverse outcomes, including complications, readmissions, reoperations, and death, increased progressively with both advancing age and higher ASA class.

In summary, among octogenarians, the rates of complications ranged from 6% to 12%, non-home discharge from 6% to 22%, readmission from 3% to 11%, reoperation from 3.5% to 4.5%, and mortality from 0.5% to 2.0%, depending on comorbidity burden. Corresponding rates were markedly lower in younger age groups, with disproportionately growing differences in mortality and non-home discharge observed among patients with severe comorbidity (ASA 4–5).

**Fig. 3:**
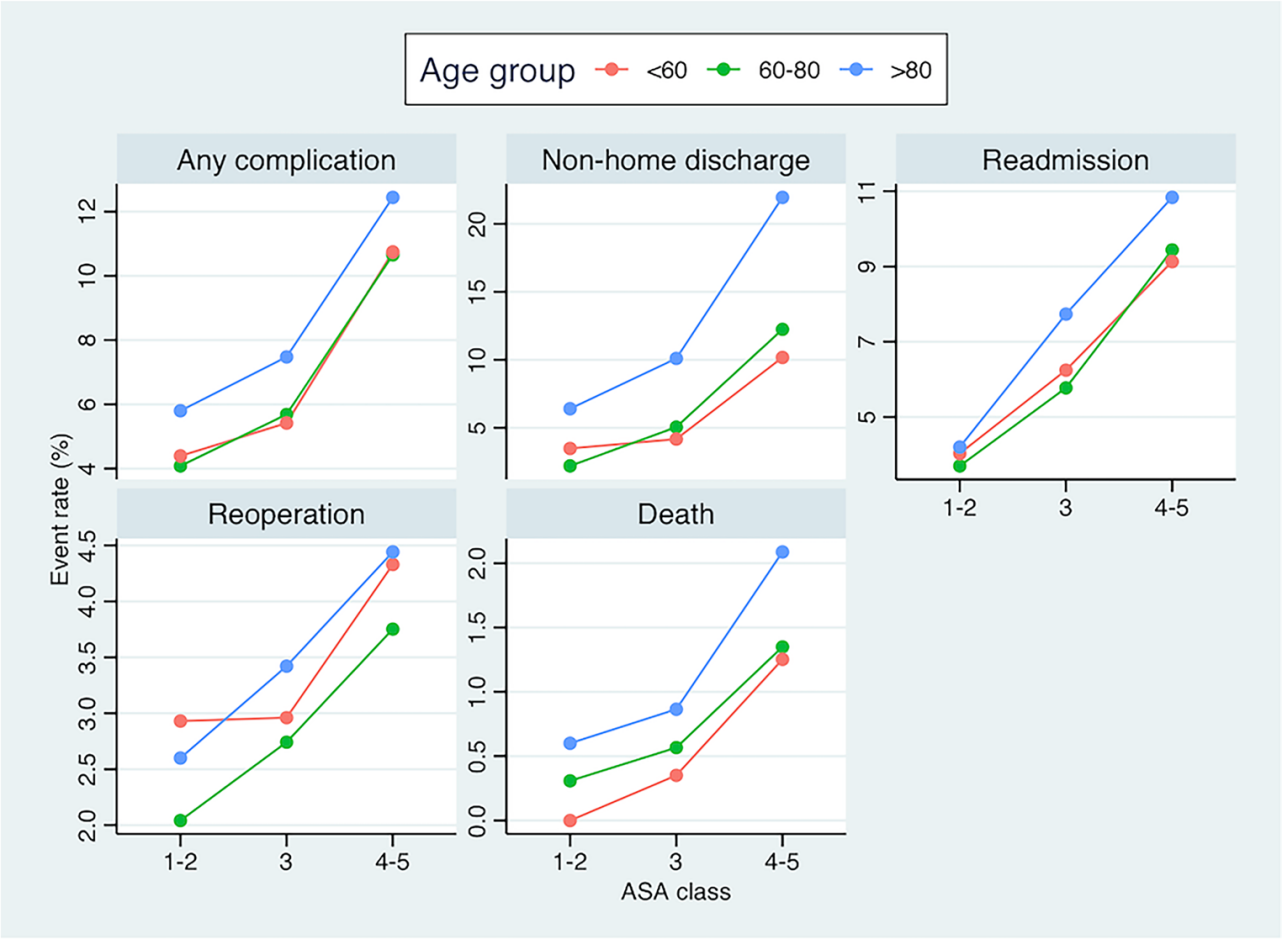
Interaction plot illustrating the combined impact of age and ASA class on 30-day outcomes following CEA

As illustrated in the interaction plot (Fig. 3), octogenarians with low comorbidity burden (ASA 1–2) demonstrated a comparable risk of several of the adverse outcomes, to younger patients with an intermediate comorbidity burden (ASA 3). In contrast, patients with severe comorbidity (ASA 4–5) experienced substantially higher rates of adverse outcomes in all age groups. Octogenarians with an intermediate burden of comorbidity (ASA 3) demonstrated lower risks of adverse outcomes across all metrics, compared to younger patients with severe comorbidity (ASA 4–5).

## Discussion

In a large nationwide dataset, older age was associated with increased risks of adverse outcomes following CEA. A significantly higher rate of most complications, non-home discharges, readmissions, reoperations, and deaths were seen in the octogenarian population, both prior to and after propensity score matching (*p* < 0.001). Notably, while patients in both < 60 and 60–80 year groups demonstrated similar risks of adverse outcomes (*p* = 0.13), octogenarians (> 80) demonstrated 34% increased odds of adverse outcomes (aOR = 1.34; *p* < 0.001).

The question of whether CEA should be considered a high-risk procedure and avoided in the octogenarian population [14], ultimately hinges on what level of adverse outcome risk is deemed acceptable relative to the potential benefits of the procedure. Although the registry-based design of the present study precludes assessment of the long-term benefits of CEA in this population, some studies have begun to broach the topic.

In one study by Fahad et al., the authors analyzed the long-term freedom from stroke as well as overall survival in octogenarians undergoing CEA. At 5-years postoperatively, the overall survival rate was 61%, with about 92% of patients being free from strokes [9]. Similarly, a Japanese study by Okawa et al. did not report any late stroke or stroke-related mortality after CEA in octogenarians during the 5-year follow-up period [16]. Additionally, the authors found no significant difference in overall survival between octogenarians and non-octogenarians (*p* = 0.371) [16]. In another study on octogenarians, Ting et al. reported a 4-year rate of freedom from stroke rate of 94% [21]. 

In a study by Ballotta et al. comparing octogenarians undergoing CEA combined with best medical treatment versus best medical treatment alone, CEA with best medical treatment demonstrated a significant advantage in terms of freedom from stroke at 5 years (98% vs. 84%; *p* = 0.04) [2]. However, there were no differences in 5-year overall survival between the groups (66% vs. 68%; *p* = 0.65). It is worth mentioning that the study by Ballotta et al. only included asymptomatic octogenarians [2]. In a large meta-analysis including 47 studies on 107,587 patients, Leung et al. found similar 1-year survival rates (95% vs. 97.5%; *p* = 0.08) and 5-year stroke risks (11.9% vs. 12.8%; *p* = 0.24) among octogenarians and non-octogenarians, respectively [11]. 

On another note, the overall risk of complications following CEA in octogenarians has been consistently reported as relatively low across the literature, including the current study. In this study, the 30-day rate of complication was 6–7.5.5% for octogenarians with low to moderate comorbidity burdens (ASA 1–2 and 3), and 12% for those with severe comorbidity burdens (ASA 4–5). The corresponding rates for younger patients ranged between 4 and 6% for patients with low to moderate comorbidity burdens (ASA 1–2 and 3), and 11% for patients with severe comorbidity burdens (ASA 4–5). This underscores the fact that only minor age-related differences in terms of short-term outcomes are available in patients with low to moderate comorbidity burdens. On that note, in the meta-analysis by Leung et al., the authors only found a modest increase in perioperative risk with age in symptomatic patients undergoing carotid endarterectomy [11]. Among other perioperative events, cardiovascular complications, particularly myocardial infarction, remain among the most feared [3, 10, 21]. Notably, one prior study demonstrated that elderly patients in particular may benefit from a reduced risk of myocardial infarction when CEA is performed under locoregional rather than general anesthesia [6]. 

In summary, evidence on long-term outcomes suggests that octogenarians can indeed derive benefit from CEA, with improvements in both stroke prevention and overall survival. These findings support the notion that a modestly higher perioperative risk may be acceptable, particularly in patients with low comorbidity burden, and potentially in those with intermediate comorbidity when selected with greater caution. Conversely, the disproportionately elevated 30-day risks of complications, non-home discharge, readmission, reoperation, and particularly mortality among octogenarians with severe comorbidity (ASA 4–5) or functional dependency may represent an absolute contraindication to surgery.

Importantly, in the current study, comorbidity burden and functional status emerged as even stronger predictors of short-term adverse outcomes when compared to age. Patients with severe comorbidity burdens (ASA 4–5; aOR = 2.17; *p* < 0.001) or partial (aOR = 1.64; *p* < 0.001) and total dependency (aOR = 2.61; *p* < 0.001) exhibited adjusted odds ratios exceeding those associated with age alone (aOR = 1.34; *p* < 0.001). This underscores the fact that physiological reserve and baseline health status exert a greater influence on postoperative risk than chronological age per se. The short-term risk associated with undergoing CEA as an octogenarian is comparable to that conferred by having a moderate comorbidity burden (ASA class 3), with both independently being associated with a 34% increase in the odds of 30-day adverse outcomes. In contrast, the general risk associated with operating on an octogenarian is substantially lower than that observed in patients with severe comorbidity (ASA 4–5) or significant functional dependency. On par with these findings, recent studies have highlighted frailty, reflecting the overall state of health of a patient, as a more robust predictor of operative morbidity and mortality following CEA, when compared to either age or comorbidity alone [8, 17, 19]. In a recent model for the prediction of adverse outcomes following CEA, developed by Eslami et al., the single strongest predictor of adverse outcomes was admission from a nursing home, with these patients experiencing a 7.6-fold increase in odds of adverse outcomes [8]. These results are consistent across the literature [12, 13, 23]. Collectively, these findings highlight the central role of frailty and baseline functional dependence on surgical outcomes, beyond the isolated impact of chronological age.

Age alone should not determine whether surgery is considered “high risk”; rather, comorbidity burden, functional dependence as a marker of frailty, and chronological age should be collectively weighed in the surgical decision-making. While CEA surgery is relatively safe in octogenarians with low to moderate burden of comorbidity (ASA 1–2 and 3), it is less so in octogenarians with severe comorbidity burdens (ASA 4–5). In this category of patients, carotid angioplasty and stenting may be better suited, pending further research.

### Limitation

Our study is subject to the limitations inherent to retrospective and registry-based studies. Although propensity score matching was used, the risk of confounding bias due to variables that are unaccounted for in the NSQIP database still exists. Some of the variables that were unavailable include surgeon’s experience, and operating center’s case volume, potentially introducing bias. A key limitation of the NSQIP dataset, in this context, is the lack of detail regarding symptomatic status, degree of stenosis, and contralateral disease. Elderly patients and those with higher ASA class may be more likely to be symptomatic or have advanced bilateral disease, which could partially explain the higher complication and mortality rates observed. The inability to stratify outcomes by symptom status or stenosis severity limits direct comparison with established treatment thresholds and warrants cautious interpretation of these findings. Finally, this study only focused on short-term outcomes, as long-term outcome data was absent.

## Conclusion

In this large nationwide analysis, older age (> 80) was associated with increased short-term (30-days) adverse outcomes following CEA, with octogenarians experiencing higher rates of complications, readmission, reoperation, non-home discharge, and mortality compared with younger patients (< 60 and 60–80). However, comorbidity burden and functional dependency emerged as stronger predictors of postoperative risk when compared to age alone. Patients with severe comorbidity (ASA 4–5) or functional dependency had substantially higher odds of adverse outcomes, while octogenarians with low to moderate comorbidity demonstrated only modestly increased risks. These findings underscore that physiological reserve and baseline health status, rather than chronological age alone, should guide surgical decision-making. CEA can be performed safely in carefully selected octogenarians, particularly those with low to moderate comorbidity burden, whereas severe comorbidity or functional dependency may represent relative contraindications. Ultimately, age should be considered within the broader context of frailty, comorbidity, and functional status when determining surgical candidacy.

## Supplementary Information

Below is the link to the electronic supplementary material.

Supplementary Figure A. Love plot illustrating covariate balance pre- and post-propensity score matching (PNG 144 KB)

High Resolution Image (TIFF 969 KB)

## Data Availability

The dataset can be provided upon reasonable request.
