# Supplementary figures and images for: Safety of carotid endarterectomy in the elderly and octogenarian population: a nationwide study including 80,000 patients
Source: Neurosurg Rev. 2026 Feb 14;49(1):226. doi: 10.1007/s10143-026-04174-4 (PMC12904964; doi:10.1007/s10143-026-04174-4)

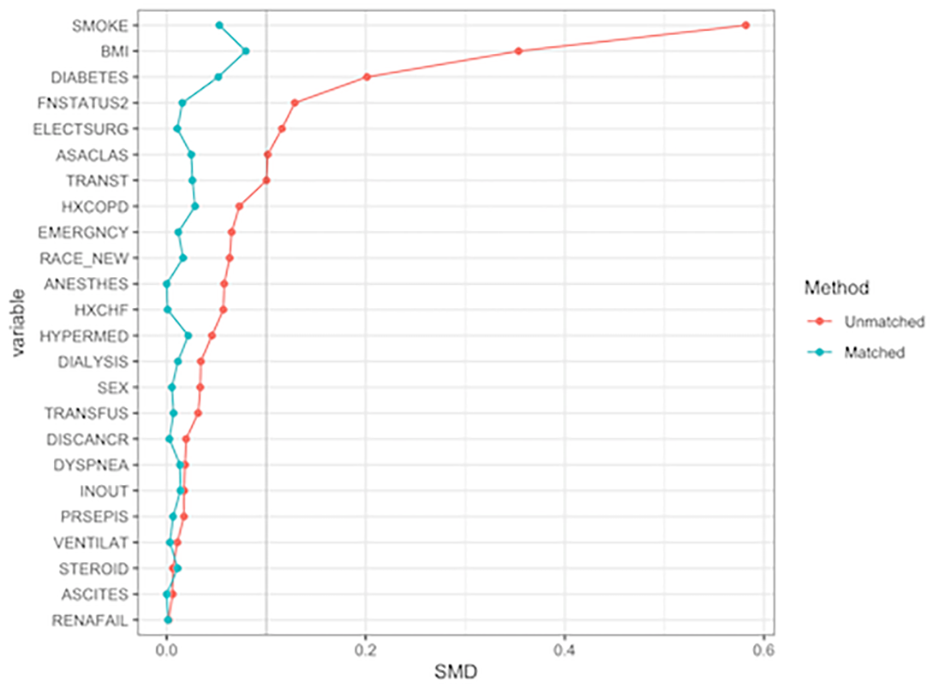

Supplement: Supplementary file 1 — Supplementary Figure A. Love plot illustrating covariate balance pre- and post-propensity score matching (PNG 144 KB) [file 10143_2026_4174_Fig4_ESM.png]

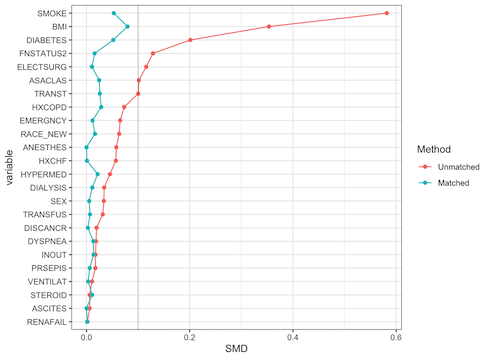

Supplement: Supplementary file 2 — High Resolution Image (TIFF 969 KB) [file 10143_2026_4174_MOESM1_ESM.tiff]
